# Non-invasive ventilation for preoxygenation before general anesthesia: a systematic review and meta-analysis of randomized controlled trials

**DOI:** 10.1186/s12871-022-01842-y

**Published:** 2022-09-30

**Authors:** Tsai-Lien Chiang, Ka-Wai Tam, Jui-Tai Chen, Chung-Shun Wong, Chun-Ting Yeh, Ting-Yun Huang, Jiann-Ruey Ong

**Affiliations:** 1 Division of Emergency Medicine, Department of Emergency and Critical Care, Taipei Medical University-Shuang Ho Hospital, 291, Zhongzheng Road, Zhonghe District, New Taipei City, 23561 Taiwan; 2 Center for Evidence-Based Health Care, Taipei Medical University-Shuang Ho Hospital, New Taipei City, Taiwan; 3 Division of General Surgery, Department of Surgery, Taipei Medical University-Shuang Ho Hospital, New Taipei City, Taiwan; 4 Division of General Surgery, Department of Surgery, School of Medicine, College of Medicine, Taipei Medical University, Taipei, Taiwan; 5 Cochrane Taiwan, Taipei Medical University, Taipei, Taiwan; 6 Department of Anesthesiology, Taipei Medical University-Shuang Ho Hospital, New Taipei City, Taiwan; 7 Department of Anesthesiology, School of Medicine, College of Medicine, Taipei Medical University, Taipei, Taiwan; 8 Department of Emergency Medicine, School of Medicine, College of Medicine, Taipei Medical University, Taipei, Taiwan

**Keywords:** Preoxygenation, Ventilation, Non-invasive positive pressure ventilation, Apnea, Desaturation, Meta-analysis

## Abstract

**Background and objectives:**

Preoxygenation is crucial for providing sufficient oxygen reservoir to a patient before intubation and enables the extension of the period between breathing termination and critical desaturation (safe apnoea time). Conventionally, face mask ventilation is used for preoxygenation. Non-invasive ventilation is a new preoxygenation method. The study objective was to compare the outcomes of non-invasive ventilation and face mask ventilation for preoxygenation.

**Method:**

PubMed, Embase, Cochrane Library, and the ClinicalTrials.gov registry were searched for eligible studies published from database inception to September 2021. Individual effect sizes were standardized, and a meta-analysis was conducted using random effects models to calculate the pooled effect size. Inclusion criteria were randomised controlled trials of comparing the outcomes of non-invasive ventilation or face mask ventilation for preoxygenation in patients scheduled for surgeries. The primary outcome was safe apnea time, and the secondary outcomes were post-operative complications, number of patients who achieved the expired O_2_ fraction (FeO_2_) after 3 min of preoxygenation, minimal SpO_2_ during tracheal intubation, partial pressure of oxygen in the arterial blood (PaO_2_) and partial pressure of carbon dioxide (PaCO_2_) after preoxygenation, and PaO_2_ and PaCO_2_ after tracheal intubation.

**Results:**

13 trials were eligible for inclusion in this study. Significant differences were observed in safe apnoea time, number of patients who achieved FeO_2_ 90% after preoxygenation for 3 min, and PaO_2_ and PaCO_2_ after preoxygenation and tracheal intubation. Only in the non-obese subgroup, no significant difference was observed in safe apnoea time (mean difference: 125.38, 95% confidence interval: − 12.26 to 263.03).

**Conclusion:**

Non-invasive ventilation appeared to be more effective than conventional methods for preoxygenation. We recommend non-invasive ventilation based on our results.

**Supplementary Information:**

The online version contains supplementary material available at 10.1186/s12871-022-01842-y.

## Background

An unexpected difficult airway during intubation can be challenging. Insufficient oxygenation causes hypoxemia followed by failed tracheal intubation (TI); this is the main concern in general anesthesia induction. SpO_2_ < 70% can cause hemodynamic instabilities, arrhythmias, hypoxic encephalopathy, and even death [1]. However, difficult TI incidence with Intubation Difficulty Scale scores of > 5, which is widely used as a cut-off value to determine moderate-to-major intubation difficulty, range from 4.5 to 11.8% [2–5].

Various factors can lead to difficult TI, such as obesity, anatomical anomaly, odontogenic infections, trauma, and limited motion range of the cervical spine or temporomandibular joints [6]. Predictable difficult TI can be managed with appropriate preparation of personnel, equipment, and the environment. However, difficult TI cannot always be predicted [7, 8]. Unanticipated difficult airway has been noted in 1.5–8.5% of anesthetized patients in clinical practice [9–11].

Preoxygenation with 100% oxygen supply may prevent hypoxemia during TI through lung denitrogenation and plasma oxygenation [12]. This enables the extension of “safe apnea time,” which increases the tolerance threshold of patients to apnea. This technique has been proven to effectively delay desaturation during apnea after anesthesia induction [13, 14]. Positive pressure ventilation during preoxygenation through continuous positive pressure ventilation (CPAP) may be beneficial in promoting gas exchange and reducing the desaturation rate [13, 15].

In the conventional method of preoxygenation, tidal volume ventilation is provided using a bag-valve mask (BVM) manually or a nonrebreathing face mask (NRM) for supplying 100% oxygen for 3 min [16, 17]. Effective preoxygenation with BVM requires one trained personnel to provide a good mask seal against the face and a one-way valve at the exhalation port, but standard BVM does not have a one-way valve built in, and this drastically decreases the oxygen fraction, making it similar to room air ventilation [17, 18].

NRM combines a face mask and a reservoir bag with a one-way valve that prevents exhaled air from re-entering the reservoir bag [19]. NRM may provide 65–80% FiO_2_ [20]. If the NRM functions well and the mask is sealed properly, SpO_2_ may reach 90% in up to 8 min [21]. However, NRMs are usually of a free size; therefore, they do not provide a good mask seal. Mask ventilation can be difficult in people with obesity, facial anatomy anomaly, facial hair growth, lack of teeth, sunken cheeks, etc., as well as in elderly patients. Moreover, NRM malfunction may lead to carbon dioxide retention and suffocation.

Non-invasive ventilation (NIV) is a recently introduced alternative preoxygenation method. NIV settings include CPAP, bilevel positive airway pressure, and pressure support ventilation (PSV) with or without positive end-expiratory pressure (PEEP). These ventilation types may improve gas exchange, decrease breathing efforts, and reduce the chances of atelectasis [22, 23]. The face masks used for NIV have a good mask seal and provide FiO_2_ of 1.0; straps can be wrapped around the patient’s head; therefore, trained personnel is not required to secure the mask at bedside [24–28]. In critical patients with acute respiratory failure, NIV is beneficial for aiding oxygenation by unloading the respiratory muscles, recruiting alveoli, and increasing the lung volume [29]. In a previous meta-analysis involving obese (BMI ≥ 35 kg/m^2^) patients scheduled for surgeries, NIV significantly improved gas exchange before TI and resulted in increased carbon dioxide clearance, improved pulmonary function, and decreased postoperative respiratory complications [30]. Nevertheless, tight-fitting NIV masks create pressure sores over the face and nose easily [31–33]. Furthermore, NIV increases the possibility of nasal and oral congestion or dryness, eye irritation, gastric insufflation, and discomfort from positive pressure, making it undesirable from the patient’s perspective [34].

This study evaluated the benefit of using NIV for preoxygenation in both obese and nonobese patients scheduled for surgery through a systemic review and meta-analysis.

## Methods

### Selection criteria

Randomized controlled trials (RCTs) comparing the outcomes of NIV and conventional preoxygenation methods in patients scheduled for surgeries were included in this review. Studies were selected only if the inclusion and exclusion criteria for patients, preoxygenation technique, and definitions of each recorded outcome were clearly reported. We excluded trials that met at least one of the following criteria: (1) pediatric patients, (2) critically ill patients with acute respiratory failure or ventilation distress that required emergency intubation, (3) trials that only recruited healthy volunteers, (4) overlap of authors, centers, or patient cohorts in two or more trials.

### Search strategy and study selection

The PubMed, Embase, and Cochrane Library databases were searched for eligible studies published from database inception to September 2021. The following Medical Subject Headings were used: ((positive pressure) OR (non-invasive)) AND ((preoxygenation) OR (ventilation) OR (anesthesia)). The detailed search strategy is described in the supplementary files (Additional file 1: Appendix 1). The “related articles” option in PubMed was used to broaden the search, and all abstracts, trials, and citations retrieved were reviewed. In addition, we identified some relevant trials from the reference sections of relevant papers and through correspondence with subject experts. Finally, unpublished trials were collected from the ClinicalTrials.gov registry (http://clinicaltrials.gov/). No language restrictions were applied. The systematic review described herein is accepted by PROSPERO, an online international prospective register of systematic reviews curated by the National Institute for Health Research (CRD42020203173).

### Data extraction

Baseline and outcome data were independently retrieved by two reviewers (TLC and KWT), and study designs, study population characteristics, inclusion and exclusion criteria, preoxygenation techniques, and collected data outcomes were extracted. Decisions recorded individually by the reviewers were compared, and disagreements were resolved by a third reviewer (JRO). The authors of the trials were contacted for additional information.

### Appraisal of methodological quality

The reviewer independently assessed the methodological quality of each trial by using the Risk of Bias Assessment 2.0 recommended by the Cochrane Collaboration [35]. Several domains were assessed, including randomization adequacy, allocation concealment, outcome assessor blinding to patient information, follow-up duration, information provided to participants regarding trial withdrawal, whether intention-to-treat analysis was performed, and freedom from other biases. We also assess the quality of evidences by using Grading of Recommendations Assessment, Development, and Evaluation (GRADE) system (Table 3).

### Outcomes

The primary outcome was safe apnea time. The secondary outcomes included postoperative complications, number of patients who achieved the expired O_2_ fraction (FeO_2_) after 3 min of preoxygenation, minimal SpO_2_ during TI, PaO_2_ and PaCO_2_ after preoxygenation, and PaO_2_ and PaCO_2_ after TI.

### Statistical analyses

Data were analysed using Review Manager, version 5.4 (The Cochrane Collaboration, Oxford, England). This trial followed PRISMA guidelines [36]. Standard deviations were estimated from the provided confidence interval limits or standard error. For the trials that reported the median and IQR or confidence interval and standard error instead of mean and standard deviation, we converted the results to mean and estimated standard deviation by using published methods [37, 38]. Dichotomous outcomes were analyzed using risk ratios as the summary statistic. The effect sizes of continuous outcomes were reported as the weighted mean difference. The precision of the effect sizes was reported as 95% CIs. Pooled estimates of the risk ratio and weighted mean difference were computed using the DerSimonian and Laird random effects models [39].

Statistical heterogeneity and the inconsistency of treatment effects across the trials were evaluated using Cochrane Q tests and I^2^ statistics, respectively. Statistical significance was set at *p* < 0.10 for Cochrane Q tests. Statistical heterogeneity across the trials was assessed using I^2^ statistics, which quantify the proportion of the total outcome variability across the trials. Moreover, subgroup analyses were performed through the pooling of available estimates for similar subsets of patients across the trials.

## Results

### Trial characteristics

**Fig. 1:**
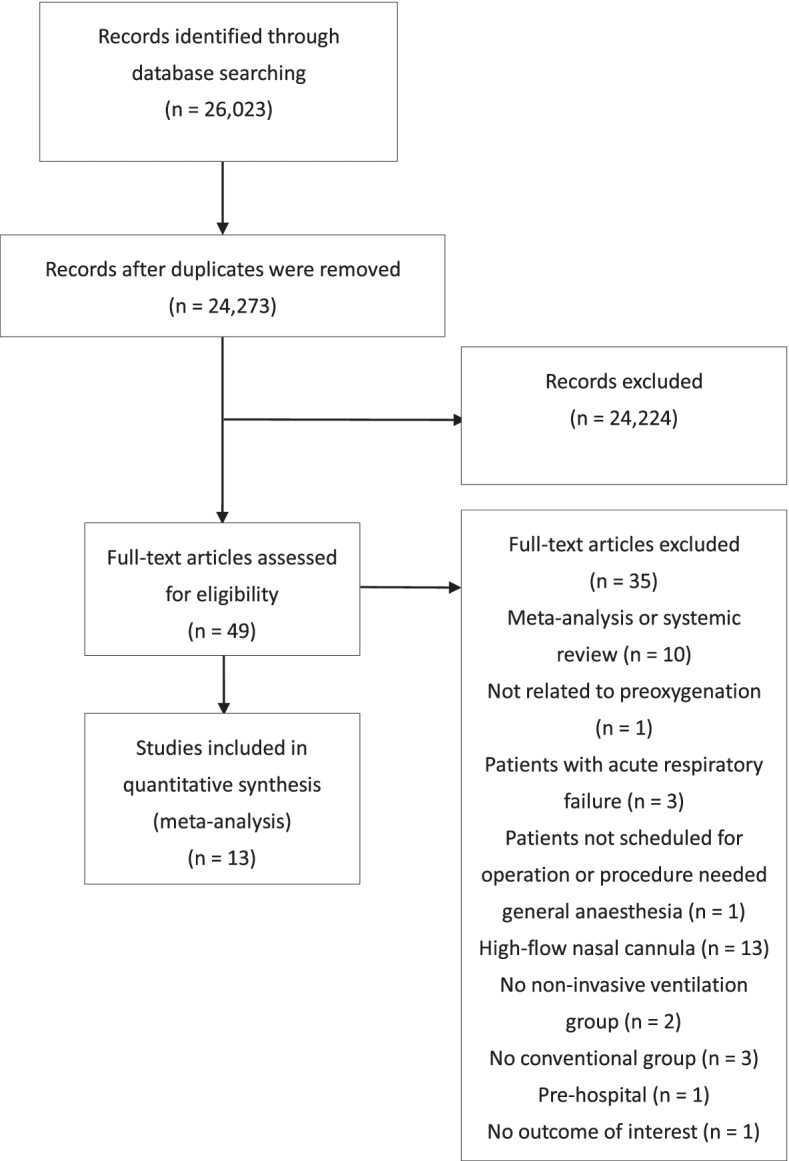
Flowchart of study selection

Figure 1 presents a flowchart of trial screening and selection. The initial search yielded 24,273 citations, of which 48 were ineligible based on the criteria used for screening titles and abstracts. Thus, the full texts of these trials were retrieved. However, most of these trials were excluded from our final review because of the following reasons: 13 used different interventions; 10 were review articles, 6 did not meet our patient selection criteria, 5 lacked control group and 1 provided no outcome of interest. Thus, 13 trials were eligible for inclusion in this study [28, 40–51].

These selected 13 trials were published between 2001 and 2021 and had sample sizes ranging from 18 to 146. Most trials recruited patients undergoing elective surgery, including bariatric surgery and neurosurgery. One trial recruited patients undergoing ear, nose, and throat panendoscopy instead of elective surgery [40]. Ten trials evaluated obese patients with BMI ≥ 30 kg/m^2^ [28, 41–43]. The other three trials evaluated nonobese patients [44–46]. The patients of every control group in the included trials were administered 100% oxygen with spontaneous breathing. Although ventilator settings in the conventional technique groups varied in terms of the ventilation mode, airway pressure, PEEP pressure, ventilation duration, and others across the trials, the NIV group received only NIV for preoxygenation. Of the 13 included RCTs, 10 were balanced. In one trial, significantly younger patients were included in the NIV group than in the conventional group [41]. In the two other trials, the proportion of men was more in the control group than in the NIV group (Table 1) [40, 47].

**Table 1:** Characteristics of included randomised controlled trials

Author (Year)	Study design	Inclusion criteria	Number of patients (% male)^a^	Age, year, mean ± SD	BMI, kg/m^−2^, mean ± SD	Intervention
Cressey. (2001) [41]	RCT	Age > 18 years; BMI > 35; patient received elective surgery; ASA I–III	V: 10 (0) C: 10 (0)	V: 34 ± 8 C: 47 ± 11	V: 45 ± 7.0 C: 44 ± 5.6	V: CPAP 7.5 cm H_2_O × 3 min C: Spontaneous breathing with Mapleson A breathing system 8 L/min
Herriger (2004) [46]	RCT	Age 16–60 years; BMI < 25; patient received elective surgery; ASA I–II	V: 20 (60) C: 20 (55)	V: 34 ± 13 C: 36 ± 8	V: 22 ± 2 C: 22 ± 2	V: CPAP 6 cm H_2_O with PEEP 6 cm H_2_O × 5 min C: Spontaneous breathing without CPAP or PEEP
Coussa (2004) [42]	RCT	Age 20–65 years; BMI > 35; patient received elective bariatric surgery; ASA II–III	V: 9 (22.2) C: 9 (0)	V: 41 ± 14 C: 37 ± 8	V: 42 ± 6 C: 44 ± 7	V: CPAP 10 cm H_2_O × 5 min C: Spontaneous breathing
Gander (2005) [43]	RCT	Age 18–60 years; BMI > 35; patient received elective surgery; ASA II–III	V: 12 (25) C: 15 (13.3)	V: 35 ± 8 C: 38 ± 12	V: 46 ± 7 C: 47 ± 6	V: CPAP 10 cm H_2_O × 5 min C: Spontaneous breathing
Delay (2008) [28]	RCT	Age > 18 years; BMI > 40; patient received abdominal surgery	V: 14 (21.4) C: 14 (14.3)	V: 36.6 ± 11.7 C: 42.9 ± 11.6	V: 47.1 ± 6.2 C: 52.3 ± 13.7	V: PSV 6 cm H_2_O with PEEP 4 cm H_2_O during the first 20 s, then PSV 8–10 cm H_2_O with PEEP 6 cm H_2_O to achieve VTe of 8 mL/kg × 5 min C: Spontaneous breathing
Futier (2011) [48]	RCT	Age > 18 years; BMI > 40; patient received LSG or Roux-en-Y gastric bypass; ASA II–III	V: 22 (27.2) VR: 22 (36.3) C: 22 (22.7)	V: 42 ± 10 VR: 43 ± 11 C: 41 ± 9	V: 46 ± 2 VR: 45 ± 5 C: 46 ± 4	V: PSV < 18 cm H_2_O with PEEP 6–8 cm H_2_O × 5 min VR: PSV < 18 cm H_2_O with PEEP 6–8 cm H_2_O with RM × 5 min C: Spontaneous breathing
Georgescu (2012) [50]	Crossover RCT	Age 18–75 years; BMI > 30; patient received elective surgery	V_1_ + V_2_: 30 (53.3)	V_1_ + V_2_: 49.6 ± 14.0	V_1_ + V_2_: 36.5 ± 5.3	V_1_: NIPPV 4 cm H_2_O with PEEP 4 cm H_2_O × 3 min, and then spontaneous breathing V_2_: Spontaneous breathing; then NIPPV 4 cm H_2_O with PEEP 4 cm H_2_O × 3 min
Harbut (2014) [51]	RCT	Age > 18 years; BMI > 35; patient received elective gastric bypass surgery; ASA II–III	V: 22 (N/P) C: 22 (N/P)	V: 46.9 ± 12.9 C: 42.1 ± 12.4	V: 43 ± 6.3 C: 44.1 ± 6.0	V: CPAP 5 cm H_2_O/PSV 5 cm H_2_O with PEEP 7 cm H_2_O × 2 min C: Spontaneous breathing
Hanouz (2015) [44]	RCT	Age > 18 years; patient received elective surgery; ASA I–II	V: 50 (60) Vp: 47 (46.8) C: 49 (55.1)	V: 45 ± 20 Vp: 40 ± 17 C: 45 ± 18	V: 25 ± 6 Vp: 23 ± 4 C: 25 ± 5	V: NIPPV 12 cm H_2_O with PEEP 6 cm H_2_O to obtain 90% FeO_2_ Vp: NIPPV 12 cm H_2_O without PEEP C: Spontaneous breathing
Edmark (2015) [47]	RCT	Age 24–49 years; BMI 35–50; patient received elective LGBP; ASA I–II	V: 10 (10) C: 20 (35)	V: 37 [34–45] C: 43 [37–48]	V: 42.9 [44.1–44.6] C: 38.1 [36.1–41.2]	V: CPAP 10 cm H_2_O with PEEP 10 cm H_2_O × 3 min C: Spontaneous breathing
Baltieri (2015) [49]	RCT	Age 25–55 years; BMI 40–55; patient received Roux-en-Y gastric bypass bariatric surgery through laparotomy	V: 10 (20) C: 20 (20)	V: 42 ± 11.2 C: 40.7 ± 10.6	V: 44.8 ± 2.8 C: 45.72 ± 4.08	V: BiPAP 12 cm H_2_O with PEEP 8 cm H_2_O × 1 h C: Spontaneous breathing
Sreejit (2015) [45]	RCT	Age 18–70 years; BMI < 25; patient received elective surgery; ASA I–II	V: 20 (55) C: 20 (55)	V: 42.75 ± 11.97 C: 45.65 ± 12.22	V: 20.97 ± 2.29 C: 21.01 ± 2.38	V: CPAP 5 cm H_2_O with a fixed PEEP device × 5 min C: Spontaneous breathing with the same device
Abou-Arab (2016) [40]	RCT	Age > 18 years; BMI > 35; patient received ENT pan-endoscopy; ASA I–III	V: 25 (16) C: 25 (48)	V: 58 ± 13 C: 58 ± 13	V: 23.3 ± 4.7 C: 25.1 ± 6.1	V: NIPPV 4 cm H_2_O with PEEP 4 cm H_2_O until EtO_2_ exceeded 90% C: Spontaneous breathing

^a^Mean (range)

*CPAP* Continuous positive airway pressure, *PSV* Pressure support ventilation, *VTe* Expiratory tidal volume, *NIPPV* Non-invasive positive inspiratory pressure ventilation, *RM* Recruitment manoeuvre, *LSG* Laparoscopic sleeve gastrectomy, *LGBP* Laparoscopic gastric bypass, *BiPAP* Bilevel positive airway pressure, *FeO*_*2*_ Expired O_2_ fraction, *EtO*_*2*_ End-tidal oxygen concentration, *ENT* Ear, nose, and throat, *V* non-invasive ventilation, *VR* Non-invasive ventilation with recruitment manoeuvre, *Vp* Non-invasive ventilation without positive end expiratory pressure, *N/P* Not provided, *C* Spontaneous breathing with tidal volume, *RCT* Randomised controlled trial, *PEEP* Positive end-expiratory pressure

The methodological quality of the included trials is summarized in Table 2. Table 3 showed the certainty assessment. Nine trials reported acceptable randomization methods. Outcome assessors were blinded to patient information in six trials [43, 45, 47–50]. Outcome assessors were not blinded to patient information in the other seven trials. Blinding of patients and anesthetists is difficult because the device appearance and discomfort from positive pressure ventilation render the method used obvious. The number of patients lost to follow-up was acceptable (< 20%) in all trials. Other biases were non-standardization of ventilator modes and setting variables across the trials.

**Table 2:** Methodological quality assessment of included trials

Study	D1^a^	D2^b^	D3^c^	D4^d^	D5^e^	Overall
2001 Cressey	SC^f^	L^g^	L	L	L	L
2004 Herriger	SC	L	L	L	L	L
2004 Coussa	SC	SC	SC	L	L	SC
2005 Gander	SC	H^h^	SC	L	L	H
2008 Delay	L	L	L	L	L	L
2011 Futier	L	L	L	L	L	L
2012 Georgescu	SC	SC	SC	L	L	SC
2014 Harbut	L	L	L	L	L	L
2015 Hanouz	L	L	L	L	L	L
2015 Edmark	L	L	L	L	L	L
2015 Baltieri	L	L	L	L	L	L
2015 Sreejit	L	L	L	L	L	L
2016 Abou-Arab	L	L	L	L	L	L

^a^D1: Bias arising from the randomization process

^b^D2: Bias due to deviations from intended interventions

^c^D3: Bias due to missing outcome data

^d^D4: Bias in measurement of the outcome

^e^D5: Bias in selection of the reported result

^f^*SC* Some concerns, ^g^*L* Low risk, ^h^*H* High risk

**Table 3:** GRADE assessments of certainty

Certainty assessment							№ of patients		Effect		Certainty	Importance
**№ of studies**	**Study design**	**Risk of bias**	**Inconsistency**	**Indirectness**	**Imprecision**	**Other considerations**	**V**	**C**	**Relative (95% CI)**	**Absolute (95% CI)**		
**Safe Apnea Time**												
7	randomized trials	not serious	not serious	not serious	not serious	none	147	152	-	MD **96.26 higher** (29.41 higher to 163.12 higher)	High	
**Safe Apnea Time—Obese**												
4	randomized trials	not serious	not serious	not serious	not serious	none	61	63	-	MD **66.62 higher** (2.73 higher to 130.51 higher)	High	
**Safe Apnea Time—Non-obese**												
3	randomized trials	not serious	not serious	not serious	serious^a^	none	86	89	-	MD **125.38 higher** (12.26 lower to 263.03 higher)	Moderate	
**3 min PreOx FeO2 to 90% Number—Number to FeO2 90% after 3 min preoxygenation**												
2	randomized trials	not serious	not serious	not serious	not serious	strong association	61/80 (76.3%)	41/79 (51.9%)	**OR 3.01** (1.52 to 5.96)	**246 more per 1,000** (from 102 to 346 more)	High	
**PaO2 (after PreOx)**												
7	randomized trials	not serious	serious^b^	not serious	not serious	none	123	127	-	MD **5.43 higher** (1.9 higher to 8.95 higher)	Moderate	
**PaO2 (after PreOx)—Obese**												
5	randomized trials	not serious	serious^c^	not serious	not serious	none	87	87	-	MD **4.98 higher** (0.63 higher to 9.34 higher)	Moderate	
**PaO2 (after PreOx)—Non-obese**												
2	randomized trials	not serious	serious^d^	not serious	not serious	none	36	40	-	MD **6.51 higher** (1.05 higher to 11.97 higher)	Moderate	
**After PreOx vs After ETI—After preoxygenatiotion**												
6	randomized trials	not serious	not serious	not serious	not serious	none	87	87	-	MD **0.41 lower** (0.58 lower to 0.23 lower)	High	
**After PreOx vs After ETI—After ETI**												
2	randomized trials	not serious	not serious	not serious	not serious	none	44	44	-	MD **0.28 lower** (0.59 lower to 0.03 higher)	High	
**SpO2 after PreOx + PaO2 after ETI—PaO2 (after ETI)**												
3	randomized trials	not serious	serious^e^	not serious	not serious	none	64	64	-	MD **4.42 higher** (0.17 higher to 8.67 higher)	Moderate	

*CI* Confidence interval, *MD* Mean difference, *OR* Odds ratio

**Explanations**

^a^OIS criterion met, but CI overlaps no effect (i.e. CI around RR excludes 1.0)

^b^The observed value of I2 fall into the range of 50–90% may represent substantial heterogeneity

^c^The observed value of I2 fall into the range of 50–90% may represent substantial heterogeneity

^d^The observed value of I2 fall into the range of 30–60% may represent moderate heterogeneity

^e^The observed value of I2 fall into the range of 50–90% may represent substantial heterogeneity

### Safe apnea time

**Fig. 2:**
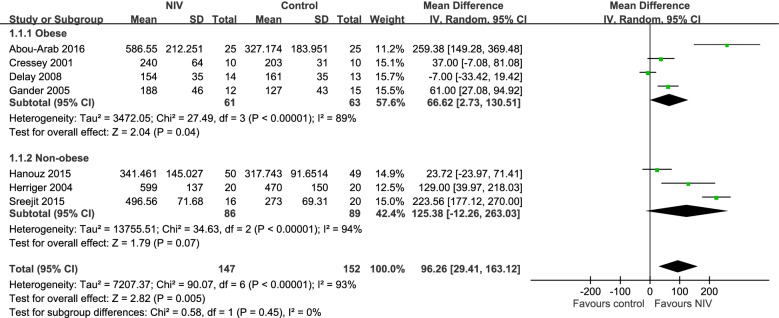
Forest plot of a comparison of safe apnea time between NIV and control groups

Seven trials compared the safe apnea time of NIV and conventional preoxygenation methods [28, 40, 41, 43–46]. Among these trials, Herriger et al., Abou-Arab et al., Cressey et al., and Gander et al. defined safe apnea time or nonhypoxemic apnea duration as the time between apnea onset and 90% SpO_2_. Hanouz et al. and Sreejit et al. defined safe apnea time as the period from apnea onset to 93% SpO_2_ [44, 45]. Delay et al. defined safe apnea time as the period from apnea onset to 95% SpO_2_ [28]. The pooled results showed that the NIV group exhibited a significantly more favorable safe apnea time than the conventional preoxygenation group (mean difference: 92.54, 95% CI: 35.31–149.78; Fig. 2).

We extracted the data of three of the seven trials with the nonobese subgroup, and no significant difference was observed between the NIV and conventional preoxygenation groups (mean difference: 125.38, 95% CI: − 12.26 to 263.03; Fig. 2).

### Incidence of people who achieved 90% FeO_2_ after 3 min of preoxygenation

**Fig. 3:**
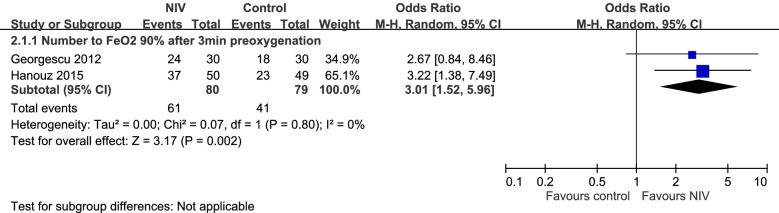
Forest plot of a comparison of number of patients who achieved FeO_2_ 90% after preoxygenation for 3 min between NIV and control groups

Two trials compared the number of patients who achieved 90% FeO_2_ through NIV and conventional preoxygenation methods [44, 50]. The NIV group achieved the favorable oxygen fraction significantly earlier than the conventional preoxygenation group (odds ratio: 3.01, 95% CI: 1.52–5.96; Fig. 3).

### Minimal SpO_2_ during TI

Only one trial reported the minimal SpO_2_ level during the TI course, and in this trial, the minimum SpO_2_ was significantly higher in the NIV group than in the control group (86.9 ± 5.0 vs 88.6 ± 2.9, mean difference − 1.70, 95% CI: − 4.73 to 1.33) [28].

### PaO_2_ after preoxygenation

**Fig. 4:**
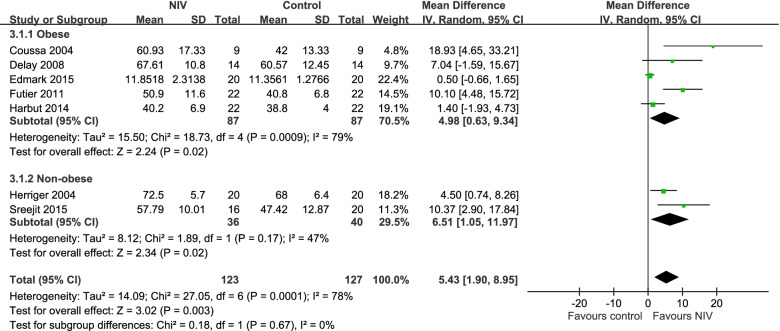
Forest plot of a comparison of PaO_2_ after preoxygenation between NIV and control groups

Seven trials compared the PaO_2_ outcome achieved after preoxygenation by using NIV and conventional methods [44, 46, 48–52]. The NIV group exhibited a significantly more favorable PaO_2_ than the conventional preoxygenation group (mean difference: 6.48, 95% CI: 2.81–10.15; Fig. 4).

After the data of both obese and nonobese groups were pooled, the results revealed a significant difference in PaO_2_ after preoxygenation between nonobese individuals in the NIV group and conventional preoxygenation group (mean difference: 6.48, 95% CI: 2.81–10.15; Fig. 4). The study population was divided into obese and nonobese subgroups; the outcomes of obese and nonobese individuals in the NIV group were significantly more favorable than those of the individuals in the conventional preoxygenation group (obese: mean difference: 4.98, 95% CI: 0.63–9.34; non-obese: mean difference: 8.42, 95% CI: 3.13–13.72; Fig. 4).

### PaCO_2_ after preoxygenation

**Fig. 5:**
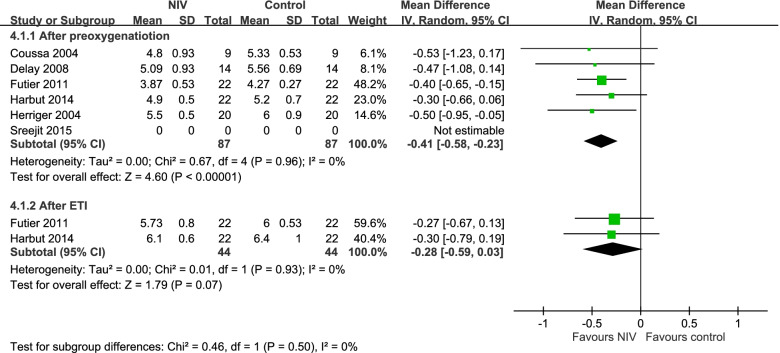
Forest plot of a comparison of PaCO_2_ after preoxygenation and after ETI between NIV and control groups

Five trials compared the PaCO_2_ outcome after preoxygenation between the NIV and conventional groups [28, 42, 46, 48, 51]. The NIV group exhibited a significantly lower PaCO_2_ than the conventional preoxygenation group (mean difference: − 0.41, 95% CI: − 0.58 to − 0.23; Fig. 5).

### PaO_2_ after TI

**Fig. 6:**
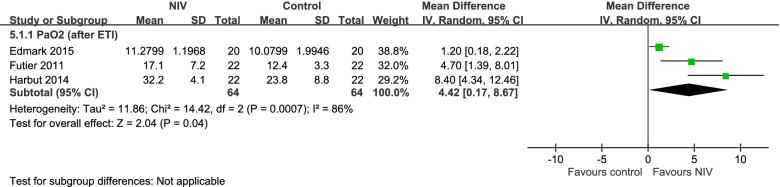
Forest plot of a comparison of PaO_2_ after ETI between NIV and control groups

Three trials compared the PaO_2_ outcome after TI between the NIV and conventional groups [47, 48, 51]. The NIV group exhibited a significantly higher PaO_2_ than the conventional preoxygenation group (mean difference: 4.42, 95% CI: 0.17–8.67; Fig. 6) after TI.

### PaCO_2_ after TI

Two trials compared the PaCO_2_ outcome after TI between the NIV and conventional groups [48, 51]. Although the NIV group appeared to have a lower PaCO_2_ than the conventional preoxygenation group after TI, the trend was not statistically significant (mean difference: − 0.28, 95% CI: − 0.59 to 0.03; Fig. 4).

### Complications

Two trials reported complications [28, 50]. Delay et al. reported that two patients (14%) in the NIV group experienced air leakage from the face mask. Furthermore, gastric distention increased to a modest degree in the NIV group compared with the low degree in the spontaneous ventilation group (3.8 ± 5.6 vs 17.6 ± 13.5, *p* = 0.01; the surgeon blinded to the oxygen administration method rated the outcome using a scale ranging from 0 [no distension] to 100 [maximal distension]). Georgescu et al. reported that one patient (7%) in the NIV group was intolerant to discomfort. Otherwise, no significant side effect was observed in either preoxygenation technique.

## Discussion

Our study found a significant difference in safe apnea time, number of patients achieving FeO_2_ after 3 min of preoxygenation, minimal SpO_2_ during TI, PaO_2_ after preoxygenation, PaCO_2_ after preoxygenation, and PaO_2_ after TI between the NIV and conventional groups. Only SpO_2_ after preoxygenation and PaCO_2_ after TI showed no significant difference, but a trend favoring NIV over conventional preoxygenation methods was found. Although the pooled results and obese subgroup showed that the NIV group exhibited a significantly more favorable safe apnea time than the conventional preoxygenation group, the extracted the data of three of the seven trials with the nonobese subgroup which also include patients with potential difficult airway intubation showed no significant difference of safe apnea time between the NIV and conventional preoxygenation groups. The results showed the possibility of NIV as an expecting method of preoxygenation, but more research is needed to determine NIV is the better preoxygenation method or not.

Spontaneous positive-pressure ventilation was first proposed experimentally as early as in the 1930s for patients with pulmonary edema [53, 54]. Later trials reported its application in patients with respiratory failure and for post-extubation respiratory rescue, facilitation of weaning, and treatment of various lung injuries [52, 55, 56]. Caples et al. (2005) reported that critical care settings favored NIV, especially for chronic obstructive pulmonary disease and acute cardiogenic pulmonary edema [57]. The trials using NIV for preoxygenation started two decades ago.

Ventilator settings across the trials were different not only in the mode chosen but also in the inspiratory pressure and volume parameters. Most trials in our study conducted CPAP and three trials conducted PSV, and both modes are commonly used in NIV practice. All the trials reported NIV to be more efficient than conventional methods for preoxygenation irrespective of the mode chosen. Regarding patient’s degree of discomfort, PSV is generally considered a more comfortable method than volume-controlled modes.

A consensus is lacking for the application of preoxygenation with PEEP. Early trials reported that PEEP may reduce atelectasis risk during anesthesia induction but may not be effective in all patients [58]. A similar problem was observed in the seven trials in which PEEP was applied in the NIV group, but comparison with an NIV group without PEEP was lacking in these trials. Generally, the NIV group, with or without PEEP, showed more favorable results than the control group in our study. Further studies are needed to confirm this statement.

A consensus is lacking for recruitment maneuver (RM) application. RM transiently increases transpulmonary pressure and thus reopens alveolar units [59]. Pulmonary RM is useful in preventing anesthesia-induced atelectasis and, thus, may aid in oxygenation in obese patients [60, 61]. An RCT included in our study (Futier et al.) reported that RM improved gas exchange and the end-expiratory lung volume, which may be associated with increased alveolar recruitment. In conclusion, RM may be helpful for preoxygenation, but more trials are needed to prove its feasibility.

In our study, 10 trials assessed the obese population, which generally experience difficulty with mask ventilation and TI [22, 62]. Gander et al. concluded that safe apnea time and BMI were negatively correlated (*r* = 0.711, *p* = 0.003) when CPAP or PEEP was not applied. In obese patients, a more effective preoxygenation method is required for safe anesthesia and intubation experiences. Our subgroup analysis showed that NIV is more beneficial than conventional methods in obese people.

Heterogeneity was found for the trials included in our study because of differences in factors such as age, sex, BMI, NIV settings, and surgical or procedural intervention. First, the preoxygenation duration differed across the trials, ranging from 2 min to unsolidified length to 90% FeO_2_ or end-tidal oxygen concentration [40, 44]. The setting of the preoxygenation time is not fixed in non–time-limited scenarios compared with the preoxygenation time for critically ill patients. The reasonable length of preoxygenation theoretically depends on the time needed to achieve denitrogenation of the functional residual capacity. Both 3 min of tidal breathing and taking eight deep breaths within 1 min have been reported to be sufficient for noncritical nonobese patients to achieve this goal [63, 64]. In our study, most included trials set the criteria as 3 or 5 min. Moreover, the control group differed among the trials due to different choices of the conventional preoxygenation method, such as ventilator facial mask, NRM, or other breathing circuit sets. Even the cut-off values of some parameters were different between the trials.

### Limitations

Our study has some limitations. First, most of the included trials had a small sample size per treatment group. Second, some outcome data provided were inadequate for pooled analysis. For example, most trials did not provide the nadir SpO2 during intubation. Futier et al. provided arterial-to-end-tidal partial pressure of carbon dioxide after 5 min of mechanical ventilation. We had anticipated that more data on postoperative performance and unplanned ICU admission would be available, but this was not the case. Third, the definitions of variables, such as the cut-off value of desaturation for safe apnea time, were different among the trials, which may limit the comparison in our study. Fourth, the assessments of air leakage from the mask, patient comfort, and additional costs associated with devices including face pads for improving sealing and reducing skin irritation were difficult to integrate. Finally, we did not include critically ill patients, children, healthy volunteers, patients with distorted head and neck anatomy, and other types of patients; thus, extending our results to these patient groups is difficult.

Tests for funnel plot asymmetry for meta-analysis should include at least 10 studies, but we don’t have more than 10 included studies available for each results, so we did not perform testing for funnel plot asymmetry [35].

## Conclusions

Our study results suggest that for preoxygenation, NIV is possibly more beneficial than conventional methods, especially in obese patients receiving selective surgeries. But for the nonobese population, we state that further studies are needed to assess whether NIV is superior to conventional method. More gastric leakage and intolerance were observed in some NIV groups, so the safety of NIV technique is a concern and may need to be further investigated.

## Supplementary Information

**Additional file 1:** **Appendix 1.** The detailed search strategy.

**Additional file 2.**

## Data Availability

All data generated or analysed during this study are included in this published article [and its supplementary information files].

## Abbreviations

FeO_2_: Oxygen fraction
PaO_2_: Partial pressure of oxygen in the arterial blood
PaCO_2_: Partial pressure of carbon dioxide
TI: Tracheal intubation
CPAP: Continuous positive pressure ventilation
BVM: Bag-valve mask
NRM: Non-rebreathing face mask
NIV: Non-invasive ventilation
PSV: Pressure support ventilation
PEEP: Positive end-expiratory pressure
RCT: Randomized controlled trial
RM: Recruitment maneuver
