# Supplementary material for: Non-invasive ventilation for preoxygenation before general anesthesia: a systematic review and meta-analysis of randomized controlled trials
Source: BMC Anesthesiol. 2022 Sep 30;22:306. doi: 10.1186/s12871-022-01842-y (PMC9524013; doi:10.1186/s12871-022-01842-y)
Supplement: Supplementary file 1 — Additional file 1: Appendix 1. The detailed search strategy. [file 12871_2022_1842_MOESM1_ESM.docx]

**Appendix 1.**

**PubMed search strategy**

| Search | Query | Details |
| --- | --- | --- |
| #1 | (non-invasive) AND (ventilator) AND (preoxygenation) AND (anesthesia) | "non-invasive"[All Fields] AND ("ventilated"[All Fields] OR "ventilates"[All Fields] OR "ventilating"[All Fields] OR "ventilation"[MeSH Terms] OR "ventilation"[All Fields] OR "ventilate"[All Fields] OR "ventilations"[All Fields] OR "ventilator s"[All Fields] OR "ventilators, mechanical"[MeSH Terms] OR ("ventilators"[All Fields] AND "mechanical"[All Fields]) OR "mechanical ventilators"[All Fields] OR "ventilator"[All Fields] OR "ventilators"[All Fields] OR "ventillation"[All Fields]) AND ("preoxygenate"[All Fields] OR "preoxygenated"[All Fields] OR "preoxygenation"[All Fields]) AND ("anaesthesia"[All Fields] OR "anesthesia"[MeSH Terms] OR "anesthesia"[All Fields] OR "anaesthesias"[All Fields] OR "anesthesias"[All Fields]) |
| #2 | (non-invasive ventilation) OR (preoxygenation) AND (anesthesia) | ("noninvasive ventilation"[MeSH Terms] OR ("noninvasive"[All Fields] AND "ventilation"[All Fields]) OR "noninvasive ventilation"[All Fields] OR ("non"[All Fields] AND "invasive"[All Fields] AND "ventilation"[All Fields]) OR "non invasive ventilation"[All Fields] OR ("preoxygenate"[All Fields] OR "preoxygenated"[All Fields] OR "preoxygenation"[All Fields])) AND ("anaesthesia"[All Fields] OR "anesthesia"[MeSH Terms] OR "anesthesia"[All Fields] OR "anaesthesias"[All Fields] OR "anesthesias"[All Fields]) |
| #3 | (positive pressure ventilation) OR (preoxygenation) AND (anesthesia) | ("positive pressure respiration"[MeSH Terms] OR ("positive pressure"[All Fields] AND "respiration"[All Fields]) OR "positive pressure respiration"[All Fields] OR ("positive"[All Fields] AND "pressure"[All Fields] AND "ventilation"[All Fields]) OR "positive pressure ventilation"[All Fields] OR "intermittent positive pressure ventilation"[MeSH Terms] OR ("intermittent"[All Fields] AND "positive pressure"[All Fields] AND "ventilation"[All Fields]) OR "intermittent positive pressure ventilation"[All Fields] OR ("positive"[All Fields] AND "pressure"[All Fields] AND "ventilation"[All Fields]) OR ("preoxygenate"[All Fields] OR "preoxygenated"[All Fields] OR "preoxygenation"[All Fields])) AND ("anaesthesia"[All Fields] OR "anesthesia"[MeSH Terms] OR "anesthesia"[All Fields] OR "anaesthesias"[All Fields] OR "anesthesias"[All Fields]) |
| #4 | ((positive pressure) OR (non-invasive)) AND (preoxygenation) AND (anesthesia) | ((("positive"[All Fields] OR "positively"[All Fields] OR "positiveness"[All Fields] OR "positives"[All Fields] OR "positivities"[All Fields] OR "positivity"[All Fields]) AND ("pressure"[MeSH Terms] OR "pressure"[All Fields] OR "pressures"[All Fields] OR "pressure s"[All Fields] OR "pressurisation"[All Fields] OR "pressurised"[All Fields] OR "pressuriser"[All Fields] OR "pressurization"[All Fields] OR "pressurizations"[All Fields] OR "pressurize"[All Fields] OR "pressurized"[All Fields] OR "pressurizer"[All Fields] OR "pressurizes"[All Fields] OR "pressurizing"[All Fields])) OR "non-invasive"[All Fields]) AND ("preoxygenate"[All Fields] OR "preoxygenated"[All Fields] OR "preoxygenation"[All Fields]) AND ("anaesthesia"[All Fields] OR "anesthesia"[MeSH Terms] OR "anesthesia"[All Fields] OR "anaesthesias"[All Fields] OR "anesthesias"[All Fields]) |
| #5 | ((positive pressure) OR (non-invasive)) AND ((preoxygenation) OR (anesthesia)) | ((("positive"[All Fields] OR "positively"[All Fields] OR "positiveness"[All Fields] OR "positives"[All Fields] OR "positivities"[All Fields] OR "positivity"[All Fields]) AND ("pressure"[MeSH Terms] OR "pressure"[All Fields] OR "pressures"[All Fields] OR "pressure s"[All Fields] OR "pressurisation"[All Fields] OR "pressurised"[All Fields] OR "pressuriser"[All Fields] OR "pressurization"[All Fields] OR "pressurizations"[All Fields] OR "pressurize"[All Fields] OR "pressurized"[All Fields] OR "pressurizer"[All Fields] OR "pressurizes"[All Fields] OR "pressurizing"[All Fields])) OR "non-invasive"[All Fields]) AND ("preoxygenate"[All Fields] OR "preoxygenated"[All Fields] OR "preoxygenation"[All Fields] OR ("anaesthesia"[All Fields] OR "anesthesia"[MeSH Terms] OR "anesthesia"[All Fields] OR "anaesthesias"[All Fields] OR "anesthesias"[All Fields])) |
| #6 | ((positive pressure) OR (non-invasive)) AND ((preoxygenation) OR (ventilation) OR (anesthesia)) | ((("positive"[All Fields] OR "positively"[All Fields] OR "positiveness"[All Fields] OR "positives"[All Fields] OR "positivities"[All Fields] OR "positivity"[All Fields]) AND ("pressure"[MeSH Terms] OR "pressure"[All Fields] OR "pressures"[All Fields] OR "pressure s"[All Fields] OR "pressurisation"[All Fields] OR "pressurised"[All Fields] OR "pressuriser"[All Fields] OR "pressurization"[All Fields] OR "pressurizations"[All Fields] OR "pressurize"[All Fields] OR "pressurized"[All Fields] OR "pressurizer"[All Fields] OR "pressurizes"[All Fields] OR "pressurizing"[All Fields])) OR "non-invasive"[All Fields]) AND ("preoxygenate"[All Fields] OR "preoxygenated"[All Fields] OR "preoxygenation"[All Fields] OR ("ventilated"[All Fields] OR "ventilates"[All Fields] OR "ventilating"[All Fields] OR "ventilation"[MeSH Terms] OR "ventilation"[All Fields] OR "ventilate"[All Fields] OR "ventilations"[All Fields] OR "ventilator s"[All Fields] OR "ventilators, mechanical"[MeSH Terms] OR ("ventilators"[All Fields] AND "mechanical"[All Fields]) OR "mechanical ventilators"[All Fields] OR "ventilator"[All Fields] OR "ventilators"[All Fields] OR "ventillation"[All Fields]) OR ("anaesthesia"[All Fields] OR "anesthesia"[MeSH Terms] OR "anesthesia"[All Fields] OR "anaesthesias"[All Fields] OR "anesthesias"[All Fields])) |

**Embase search strategy**

| Search | Query |
| --- | --- |
| #1 | 'non-invasive preoxygenation anesthesia' OR ('non invasive' AND ('preoxygenation'/exp OR preoxygenation) AND ('anesthesia'/exp OR anesthesia)) |
| #2 | 'non-invasive preoxygenation anesthesia ventilator' OR ('non invasive' AND ('preoxygenation'/exp OR preoxygenation) AND ('anesthesia'/exp OR anesthesia) AND ('ventilator'/exp OR ventilator)) |
| #3 | 'non-invasive preoxygenation anesthesia ventilation oxygenation' OR ('non invasive' AND ('preoxygenation'/exp OR preoxygenation) AND ('anesthesia'/exp OR anesthesia) AND ('ventilation'/exp OR ventilation) AND ('oxygenation'/exp OR oxygenation)) |
| #4 | 'positive pressure preoxygenation anesthesia ventilation oxygenation' OR (positive AND ('pressure'/exp OR pressure) AND ('preoxygenation'/exp OR preoxygenation) AND ('anesthesia'/exp OR anesthesia) AND ('ventilation'/exp OR ventilation) AND ('oxygenation'/exp OR oxygenation)) |
| #5 | 'positive pressure preoxygenation anesthesia oxygenation' OR (positive AND ('pressure'/exp OR pressure) AND ('preoxygenation'/exp OR preoxygenation) AND ('anesthesia'/exp OR anesthesia) AND ('oxygenation'/exp OR oxygenation)) |
| #6 | 'positive pressure non-invasive ventilator preoxygenation anesthesia oxygenation' OR (positive AND ('pressure'/exp OR pressure) AND 'non invasive' AND ('ventilator'/exp OR ventilator) AND ('preoxygenation'/exp OR preoxygenation) AND ('anesthesia'/exp OR anesthesia) AND ('oxygenation'/exp OR oxygenation)) |
| #7 | 'positive pressure non-invasive ventilation preoxygenation anesthesia' OR (positive AND ('pressure'/exp OR pressure) AND 'non invasive' AND ('ventilation'/exp OR ventilation) AND ('preoxygenation'/exp OR preoxygenation) AND ('anesthesia'/exp OR anesthesia)) |

**Cochrane Library search strategy**

| #1 | (preoxygenation ventilation anesthesia):ti,ab,kw |
| --- | --- |
| #2 | (preoxygenation ventilator anesthesia):ti,ab,kw |
| #3 | (non-invasive preoxygenation anesthesia):ti,ab,kw |
| #4 | (non-invasive positive pressure preoxygenation anesthesia):ti,ab,kw |
| #5 | (non-invasive positive pressure preoxygenation ventilation anesthesia):ti,ab,kw |
